# Quantifying and categorising national extinction-risk footprints

**DOI:** 10.1038/s41598-022-09827-0

**Published:** 2022-04-07

**Authors:** Amanda Irwin, Arne Geschke, Thomas M. Brooks, Juha Siikamaki, Louise Mair, Bernardo B. N. Strassburg

**Affiliations:** 1 ISA, School of Physics, A28, The University of Sydney, Sydney, NSW 2006 Australia; 2 IUCN, Rue Mauverney 28, 1196 Gland, Switzerland; 3 World Agroforestry Center (ICRAF), University of the Philippines Los Baños, Laguna, Philippines; 4 Institute for Marine & Antarctic Studies, University of Tasmania, Hobart, Australia; 5 IUCN, 1630 Connecticut Avenue NW, Washington, DC 20009 USA; 6 School of Natural and Environmental Sciences, Newcastle University, Newcastle upon Tyne, NE1 7RU UK; 7 Rio Conservation and Sustainability Science Centre, Department of Geography and the Environment, Pontifical Catholic University, Rio de Janeiro, Brazil; 8 International Institute for Sustainability, Rio de Janeiro, Brazil

**Keywords:** Biodiversity, Environmental economics

## Abstract

Biodiversity, essential to delivering the ecosystem services that support humanity, is under threat. Projections show that loss of biodiversity, specifically increases in species extinction, is likely to continue without significant intervention. Human activity is the principal driver of this loss, generating direct threats such as habitat loss and indirect threats such as climate change. Often, these threats are induced by consumption of products and services in locations far-removed from the affected species, creating a geographical displacement between cause and effect. Here we quantify and categorise extinction-risk footprints for 188 countries. Seventy-six countries are net importers of extinction-risk footprint, 16 countries are net exporters of extinction-risk footprint, and in 96 countries domestic consumption is the largest contributor to the extinction-risk footprint. These profiles provide insight into the underlying sources of consumption which contribute to species extinction risk, a valuable input to the formulation of interventions aimed at transforming humanity’s interactions with biodiversity.

## Introduction

The impact of consumption on biodiversity loss has been explored using a variety of proxies, such as land use, greenhouse gas emissions, and other inputs to production^1–5^, to connect species’ impacts to economic activity. The use of species’ threat information from the International Union for Conservation of Nature (IUCN) Red List of Threatened Species to connect economic activity to biodiversity loss was first explored by Lenzen et al.^6^, who found that international trade drives 30% of global species threats. Moran and Kanemoto^7^ reapplied this methodology, overlaying spatial data to uncover hotspots of species threatened by international trade. To-date, however, such environmentally-extended input–output analyses have not incorporated the wealth of species-specific information now available to assess the connection between economic activity and biodiversity loss, nor applied this methodology to quantify country-level impacts at a global scale.

The connections between final consumption and those human activities that directly impact biodiversity loss are embodied in complex global supply chains which harness, manipulate, and transform nature’s outputs into products and services, generating economic activity at each stage of the process. There are two approaches to assessing the environmental impacts of these supply chains, with production-based accounting, such as that used within the Paris Agreement^8^ which holds each country accountable for the greenhouse gases emitted within its borders, intuitive and generally straightforward. In contrast, consumption-based accounting allocates an environmental impact to the final node of each supply chain by accounting for the multitude of small impacts that accumulate as a product or service materialises along its supply chain. Although complex and computation-intensive, this accounting approach ultimately enables quantification of all consumption connections across our globalised world, where, for instance, the purchase of a new car in Europe may be a driver of land clearing in Asia.

Our consumption-based analysis utilises the input–output methodology first proposed by Leontief^9^, which considers the flow of money transacted throughout each supply chain, rather than the physical flow of materials (Supplementary Note S1). Connecting an environmental impact to these economic interactions enables that impact to be accounted for at each node of the supply chain, generating what has come to be known as a footprint, which is derived from an understanding of both the final demand for a product or service, and all the intermediate transactions required to deliver that product or service^10^.

We adopt the extinction risk of species as a quantifiable representation of biodiversity loss and present the non-normalised Species Threat Abatement and Restoration (nSTAR) metric. Calculated using detailed information from the IUCN Red List of Threatened Species^11^ for all threatened and Near Threatened terrestrial birds, mammals, and amphibians, this unit-less metric provides an additive measure of extinction risk which can be aggregated and disaggregated across the three relevant dimensions of species, country, and economic sector. When linked with the 2013 Eora multi-region input–output (MRIO) global supply chain database^12^ this metric forms the basis of the consumption extinction-risk footprint, which is further refined to derive imported, exported, and domestic extinction-risk footprints at a country level. These footprints are identified by considering the role each country serves as both a steward of the biodiversity within its borders, represented in its territorial extinction-risk footprint, and as a consumer of products whose supply chains extend beyond its borders, represented in its consumption extinction-risk footprint. The interplay between these generates a domestic footprint (the impact of a country’s consumption on extinction risk within the country), an exported footprint (the impact of other countries’ consumption on extinction risk within the country), and an imported footprint (the impact of a country’s consumption on extinction risk outside of the country) for each country (Supplementary Note S2). At a global level, or at a total species level, the territorial extinction-risk footprint and the consumption extinction-risk footprint will be equal.

## Results

### Country extinction-risk footprints

**Figure 1:**
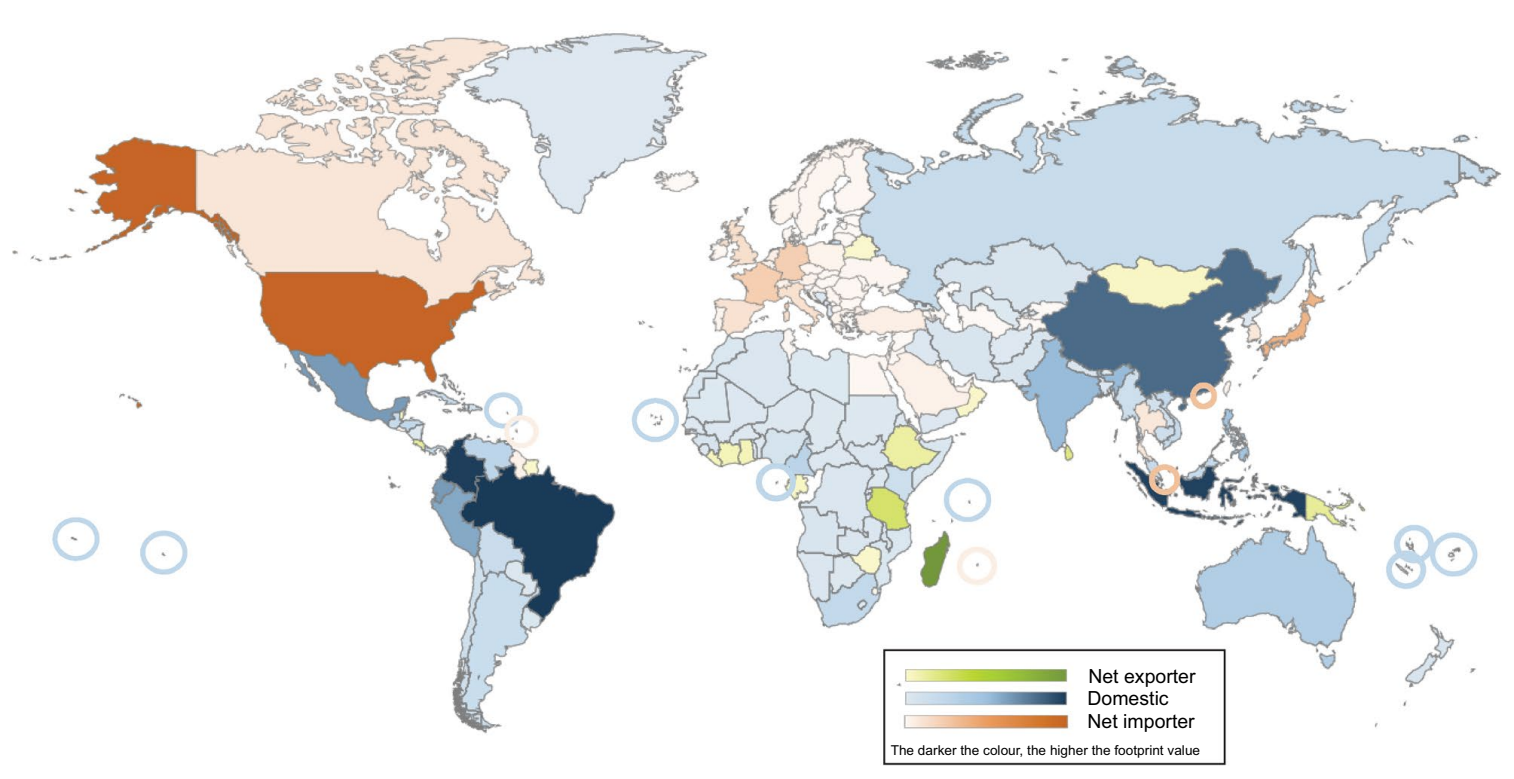
Category of extinction-risk footprint by country. Each of the 188 countries in scope is colour-coded according to which of the imported, exported, or domestic footprints has the highest value in that country. Net importers (orange) primarily drive extinction-risk footprint in other countries, the extinction-risk footprint for net exporters (green) is primarily driven by consumption in other countries, and consumption within the country primarily drives extinction-risk footprint for domestic countries (blue). The darker the colour, the higher the corresponding footprint value (imported extinction-risk footprint for countries colour-coded orange, exported extinction-risk footprint for countries colour-coded green, and domestic extinction-risk footprint for countries colour-coded blue). (Generated using Microsoft PowerPoint https://www.microsoft.com/en-us/microsoft-365/powerpoint).

**Figure 2:**
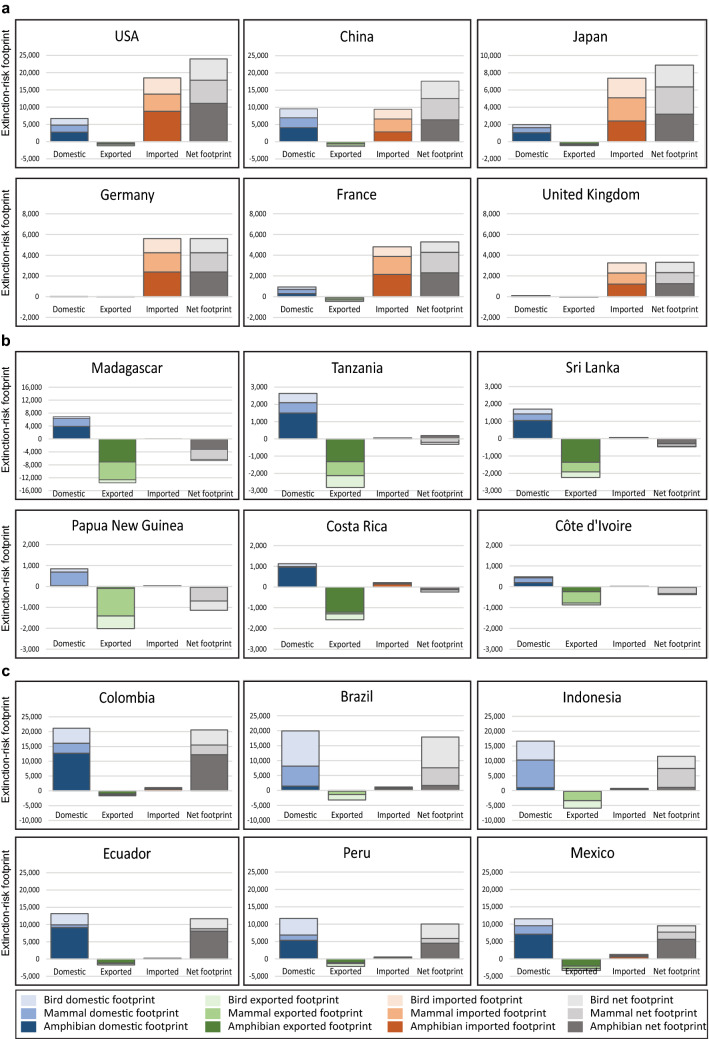
Extinction-risk profiles for selected countries. Each country’s domestic, exported, imported and net footprint is plotted for: (**a**) a selection of importers of extinction-risk footprint where, with the exception of China, the value of the imported footprint is greater than both the domestic and exported footprints; (**b**) a selection of net exporters of extinction-risk footprint, where the value of the exported footprint is greater than both the domestic and imported footprints; and (**c**) a selection of countries for which domestic consumption is the key driver of the extinction-risk footprint, with the value of the domestic footprint higher than both the exported and imported footprints. The net footprint is calculated by subtracting the exported footprint from the sum of the domestic and imported footprints. Exported footprints are plotted as negative values to reflect the fact that they are due to consumption located outside of the country in question. Note that there are no units for extinction-risk footprint.

Domestic, exported, and imported extinction-risk footprints were calculated for 188 countries, and each country was categorised according to which of the domestic, exported, or imported extinction-risk footprints was the largest. Seventy-six countries, concentrated in Europe, North America, and East Asia, are categorised as net importers of extinction-risk footprints (colour-coded orange in Fig. 1), with consumption in these countries primarily driving the extinction-risk footprint of species located in other countries. Figure 2a illustrates the net extinction-risk footprint for a selection of countries with large imported footprints and compares the footprints attributed to domestic, exported, and imported consumption in each country.

Sixteen countries, concentrated in Africa, are categorised as net exporters of extinction-risk footprints (colour-coded green in Fig. 1), with consumption based in other countries the primary driver of the extinction-risk footprint. Madagascar’s domestic consumption, for example, drives only 34% of its territorial extinction-risk footprint, with the rest driven by consumption outside of its borders led by the USA (14%), France (11%), and Germany (6%) (Dataset 2). Figure 2b illustrates the net extinction-risk footprint for a selection of these net exporters and compares the footprints attributed to domestic, exported, and imported consumption in each country. Domestic consumption is the largest component of the extinction-risk footprint for the remaining 96 countries (colour-coded blue in Fig. 1). Figure 2c illustrates the net extinction-risk footprint for a selection of these countries and compares the footprints attributed to domestic, exported, and imported consumption for each country.

Figure 2 also highlights differences in the taxonomic make-up of country extinction-risk footprints, with, for example, the majority of Colombia’s territorial footprint (60%) generated by threats to amphibians, whereas threats to birds constitute the majority of Brazil’s territorial footprint (59%). Papua New Guinea’s primary extinction risk export is mammals (65%), while Costa Rica’s is amphibians (77%). These findings are disproportionate to the underlying distribution of these taxa in Colombia (where amphibians constitute 49% of threatened or Near Threatened species), Papua New Guinea (where mammals constitute 33% of threatened or Near Threatened species), and Costa Rica (where amphibians constitute 50% of threatened or Near Threatened species). There is a relatively even spread of impact across taxonomic groups for the top importers of extinction-risk footprint, reflecting the widespread geographical sources of imports into these countries (Fig. 2a).

When the exported extinction-risk footprint is summed across all countries, we find that international trade drives 29.5% of the global extinction-risk footprint. This is consistent with previous findings which, depending on the attribute of biodiversity used to connect into economic activity, find international trade implicated in between approximately one quarter and one third of all species-driven biodiversity loss^1,6^. Our inclusion of scope and severity data for each threat rather than just the presence of that threat, and the weighting of this data by the species’ extinction risk category, enables a more extensive quantification of the extinction-risk footprint for each country, sector, and species. We can now show for example, that Madagascar’s exported extinction-risk footprint, at 66% of the total territorial extinction-risk footprint, is higher than the proportion of species threats previously linked to export using just the presence of threats (51%)^6^ and that while Indonesia, defined as a net-exporter of species threats by Lenzen et al.^6^, does indeed export more extinction-risk footprint than it imports (5875 vs 761), its domestic extinction-risk footprint (16,662) is significantly higher than either of these, led by local demand for the food crops sector.

Further detail on the size of each country’s territorial and consumption footprint, the share of its consumption footprint imported from other countries, the share of its territorial footprint exported to other countries, and the size of its per-capita consumption footprint are available in Dataset 1 at https://doi.org/10.25910/smtv-e036. This dataset also details the number of economic sectors analysed for each country and which of the country’s economic sectors generates the highest consumption extinction-risk footprint, based on consumption from all countries. Dataset 2, available at https://doi.org/10.25910/e60m-2r62, provides detail on the flow of extinction-risk footprint between countries as a percentage of each country’s territorial extinction-risk footprint. Dataset 3, available at https://doi.org/10.25910/n1sd-rw69, provides detail on the flow of extinction-risk footprint between countries as a percentage of each country’s consumption extinction-risk footprint. These detailed data provide the information each country needs to identify the key locations of consumption which drive their territorial extinction-risk footprint, and the locations of extinction-risk footprint induced by their own consumption.

### Species extinction-risk footprints

Input–output analysis can also be applied at a species level to identify the locations and sectors of consumption which are driving the extinction-risk footprint of each species. Consider the Malagasy Giant Jumping Rat (*Hypogeomys antimena*), found in Madagascar^13^. Most of the consumption linked to the extinction-risk footprint of this Endangered mammal comes from abroad, with 77% of its extinction-risk footprint exported. Further analysis reveals that 11% of its extinction-risk footprint can be traced back to demand for European food and beverage products. In western Africa, 44% of the extinction-risk footprint for the Critically Endangered Western Gorilla (*Gorilla gorilla*)^14^ is exported. In this case, consumption of China-based commodities generates 14% of the total extinction-risk footprint, with demand for the outputs of its construction sector alone generating 6% of the total footprint. The USA imports 24% of the extinction-risk footprint of the Nombre de Dios Streamside Frog (*Craugastor fecundus*), a Critically Endangered frog found in Honduras^15^, with 3% of its footprint due to consumption of USA-based tobacco, coffee, and tea commodities. Supplementary Note S3 contains more details on the locations of consumption impacting these three species.

### Sector extinction-risk footprints

Country-level consumption footprints were disaggregated by economic sector for each country, based on the expenditure data recorded against the sector of final demand, the point at which households, governments or non-profit institutions purchase a product or service. Intermediate economic transactions, such as when a food manufacturer purchases agricultural products in order to supply its food products to the final consumer, will be captured in the consumption footprint generated for the sector of final demand, in this case the food and beverage sector. Sector classifications were based on those used in each country’s economic reporting^12^, with the number of sectors ranging from 26 to 1022 sectors per country.

**Figure 3:**
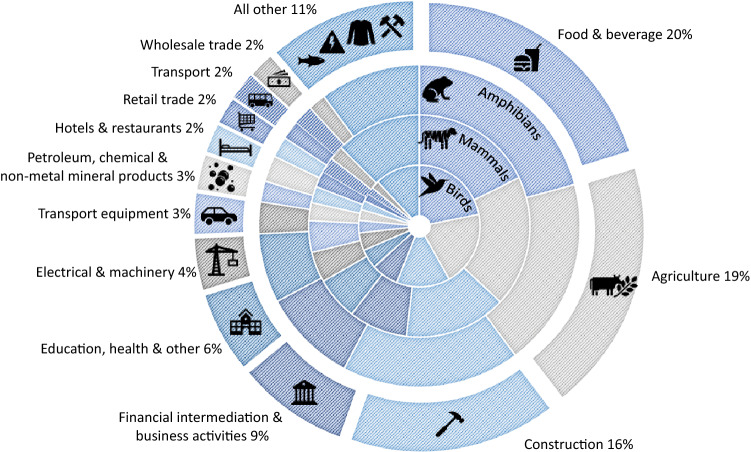
Global extinction-risk footprint by consumption sector. Each sector’s contribution to the global extinction-risk footprint is shown, including a high-level taxonomic breakdown. Country specific sector classifications have been aggregated to 26 common sectors based on the International Standard Industrial Classification. (Generated using Microsoft Excel https://www.microsoft.com/en-au/microsoft-365/excel).

In order to consolidate these country results to a global level for ease of visualisation, country-level sector classifications were aggregated to 26 common sectors based on the International Standard Industrial Classification^12,16^. This aggregation reveals that the food and beverage sector is the greatest driver of consumption-induced extinction risk globally, generating 20% of the global extinction-risk footprint, followed by the agriculture (19%) and construction (16%) sectors. This is consistent with previous sector-focused studies which have identified food consumption and agricultural activities as the most significant drivers of biodiversity loss^5,17^. Figure 3 illustrates these high-level sector contributions to global extinction-risk for all in-scope species and for each Class, with further details available in Supplementary Note S4.

Consumption of products and services provided by the food and beverage sector (or sub-sector where relevant) is the top ranked contributor to the extinction-risk footprint in 80 countries, while consumption of construction-related products and services is the top ranked contributor in 36 countries. Consumption of agriculture-related products and services is the top ranked contributor to the extinction-risk footprint in 35 countries, and another 23 countries see the greatest extinction-risk footprint generated by consumption in the education, health and other services sector (or sub-sector where relevant). The top ranked sector for each country is included in Dataset 1.

## Discussion

Our findings provide detailed information on the location and scale of the underlying sources of consumption which contribute to species extinction risk in each country, a valuable input to the formulation of policy interventions. Where there is a disconnect between the location of consumption and the resultant biodiversity loss, local interventions are likely to be less effective and international co-operation may be an important component of the policy response. Consider Colombia and Madagascar, which contribute 7% and 6.3% of the global extinction-risk footprint respectively but have very different footprint profiles. Colombia’s territorial extinction-risk footprint is driven by domestic consumption, which represents 93% of its total, while 66% of Madagascar’s territorial extinction-risk footprint is driven by consumption in other countries. Policy interventions focused on local demand, and the supply chains which support it, would seem appropriate in Colombia, while interventions to address Madagascar’s biodiversity loss may need to focus on international trading arrangements. Meanwhile, policy interventions for net importers of extinction-risk footprint, such as France, Germany, Japan, UK, and USA, may need to focus internationally to ameliorate the impacts of their consumption, for example by providing sufficient financial and capacity support to conservation and sustainable production in extinction-risk exporting countries.

The significance of the food and beverage and agriculture sectors as the highest contributors to the global extinction-risk footprint when aggregated in the sectoral dimension reinforces the importance of addressing food loss and waste, estimated at up to 33% of all food production^18^. The rationale behind current efforts to reduce this includes the contribution to global greenhouse gas emissions (estimated at 8–10% of total)^19^, water and land use^20^, eutrophication^20^, and the inherent inequity in a global society where food insecurity and food waste co-exist^21^. Adding the impact on biodiversity loss as a consideration makes the case for reducing food waste even more compelling. Success in delivering Target 12.3 of the UN’s Sustainable Development Goals, which aims for a 50% reduction in food waste at the consumer and retail levels^22^, could also deliver a reduction in the global extinction-risk footprint of more than 3%.

These findings could be used to inform the work underway by the United Nations Statistics Division to propose options for incorporating biodiversity in National Accounts^23^. The consumption footprint values calculated here in effect represent the debit against extinction risk that consumption in each country-sector combination generates. Given that these values are calculated using the sector classifications found in the System of National Accounts^12^, incorporation into the System of Environmental-Economic Accounting (SEEA) framework would be straightforward. A comprehensive measurement of the impacts of economic activities on biodiversity, such as that under development in the SEEA revision, is likely to contain an element related to species and their extinction risk, and the nSTAR metric introduced here could thus provide a way to capture ‘debit’ transactions against this measurement. Further extension of our methods, perhaps in combination with approaches such as the Spatially Explicit Information on Production to Consumption Systems model^24^ or Life Cycle Impact Assessment^4,25^, could provide insights into the consumption extinction-risk footprint associated with non-state actors such as corporations or cities. Once each sub-national actor’s contribution to national economic output is understood, each country-sector consumption extinction-risk footprint could be further allocated to this level of detail. Future research avenues could also include a study of the historical and political influences that shape the flow of extinction-risk footprints between countries, such as that seen between Madagascar and France, where 37.5% of the French consumption extinction-risk footprint is sourced from Madagascar (Dataset 3).

Patterns of global consumption, and their interconnectedness through international trade, have a significant impact on global biodiversity loss. Variations between the characteristics of each country’s extinction-risk footprint highlight the need to tailor national policy interventions cognizant of the locations of both direct impact and consumption. The use of the widely available System of National Accounts framework and IUCN Red List species assessments in the development of this methodology positions it as a meaningful contribution to the development of the post-2020 Global Biodiversity Framework under the Convention on Biological Diversity.

## Methods

Previous studies have used number of species threats^6,7^, countryside species-area relationship^1,3,17^, and potentially disappeared fraction of species^4^ to quantify biodiversity loss. We introduce the non-normalised Species Threat Abatement and Restoration (nSTAR) metric as the quantifiable representation of biodiversity loss in our analysis, a unit-less, species-centred metric which relies on detailed information curated in the IUCN Red List of Threatened Species^11^. On its own, this metric can be used to support production-based accounting of the extinction risk of species and identify the most significant threats at a specific location to inform direct interventions^26^. However, once manipulated into a structure that allows it to be appended to a multi-region input–output (MRIO) table, an environmentally-extended MRIO can be created. This unlocks the power of consumption-based accounting of this extinction risk, connecting the direct environmental impact with the consumption which ultimately induces it.

### IUCN Red List of Threatened Species

The IUCN Red List version 2020–2^11^ provided information on extinction risk for over 122,000 species and details of the threats acting on those species, including the threat classification, scope, timing, and severity. The species scope was limited to comprehensively assessed terrestrial species, ensuring that only species which have been assessed across all countries were included, and thus eliminating any geographical bias introduced by incomplete assessments^27^. Species with an extinction risk category of Near Threatened (NT), Vulnerable (VU), Endangered (EN), or Critically Endangered (CR) were included. Three species were excluded to avoid double counting where two different extinction risk categories were provided for the same species, leaving 5295 amphibian, mammal, and bird species in scope.

The information contained in the IUCN Red List regarding the threats facing each species is crucial, since many of these threats are attributable to economic activity^28,29^. Specialist assessors are required to assign one or more of 118 different threat classes to each species’ record, with additional documentation of the severity, scope and timing of each threat recommended, based on the impact of that threat on the species’ population^30^. To connect this threat information to economic sectors, a key requirement for input–output analysis, background information on threat classes was sourced from the IUCN Threats Classification Scheme version 3.2^29^. Each threat was assessed for connection to each of the 6357 economic sectors classified in the UN Statistics Division Central Product Classification Standard^31^, based on the likelihood that activity associated with each sector directly contributes to the threat being assessed. As an example, the economic sectors associated with rice cultivation were allocated to the threats grouped under IUCN Threat Class 2.1—Annual & perennial non-timber crops. A total of 55 out of 118 threats were allocated to at least one economic sector, with higher-level threat classes excluded from this allocation if information was available for the associated lower-level threat classes to avoid double counting. Species threats driven by activity that cannot be attributed to an economic sector, such as invasive species, were not allocated to any sectors and as a result, the extinction-risk footprint does not necessarily represent the full magnitude of extinction risk for each species. While not all threats were allocated to an economic sector, all economic sectors were allocated to at least one threat. Further details on the connection of economic sectors to threats are available in Supplementary Note S5, which includes a link to the detailed 6357 × 118 binary concordance matrix used to execute these sector-threat allocations.

The IUCN Red List also requires inclusion of a range map and habitat classification, which were combined with remote sensed land cover and elevation data to generate a high-resolution area of habitat (AOH) map for each in-scope species^32,33^. These maps, reapplied from Strassburg et al.^34^, were used to calculate the percentage of each species’ AOH present in each country.

### Quantifying biodiversity loss: the nSTAR metric

This detailed information from the IUCN Red List was used to calculate the nSTAR metric, which quantifies each threat’s impact, rather than just its presence, on each species. Adapted from the newly developed Species Threat Abatement and Restoration metric (STAR)^26^ by removing the normalisation step, the nSTAR metric, which has no units, was calculated for each species in two stages.

First, a numeric representation of each species’ extinction risk category (*W*_*i*_) was determined, following the equal steps methodology introduced by Butchart et al.^35^. Extinction risk categories of Data Deficient (DD) and Least Concern (LC) were assigned *W*_*i*_ = 0, Near Threatened (NT) was assigned *W*_*i*_ = 1, Vulnerable (VU) was assigned *W*_*i*_ = 2, Endangered (EN) was assigned *W*_*i*_ = 3, and Critically Endangered (CR) was assigned *W*_*i*_ = 4.

Next, a Threat Impact score *(TS*_*ij*_*)* for each threat *(j)* acting on a species (*i*) was determined based on the scope and severity information recorded for that threat, according to the values set out in Table 1, which are adapted from those proposed by Garnett et al.^36^. Reapplying the methodology of the STAR metric, where no value was recorded for the scope or severity of a threat, the median possible value for these were used, and only threats noted as Ongoing or Future were included. Further details on these methodological choices and sensitivity analyses to support them are available in Mair et al.^26^.

**Table 1:** Numeric representation of threat information.

Threat Impact score assigned	Severity of threat			
	Causing or likely to cause negligible declines *OR* No decline	Causing or likely to cause relatively slow, but significant, declines OR Causing or likely to cause fluctuations	Causing or likely to cause rapid declines (20–30% over 10 years or three generations)	Causing or likely to cause very rapid declines (> 30% over 10 years or three generations)
Scope of threat				
Affects the minority (< 50%) of the population	0	5	7	24
Affects the majority (50–90%) of the population	0	9	18	52
Affects the whole (> 90%) population	1	10	24	63

The numeric nSTAR value for each species-threat combination (*ij*) was calculated by multiplying the value representing the species’ extinction risk category (*W*_*i*_) by the Threat Impact score *(TS*_*ij*_*)* for that threat:

1 $${\text{nSTAR}}_{ij} = W_{i} *TS_{ij}$$

The total nSTAR for species *(i)* can be calculated by multiplying the extinction risk category value (*W*_*i*_) for that species by the sum of all Threat Impact scores for the species:

2 $${\text{nSTAR}}_{i} = W_{i} *(TS_{i1} + TS_{i2} + TS_{i3} + \cdots + TS_{ij} )$$

Once calculated according to Eq. (1), the nSTAR_*ij*_ value for each species-threat combination was allocated to economic sectors using the 6357 × 118 sector-threat concordance (available in Supplementary Note S5), which was normalised based on the economic size of each sector. Finally these nSTAR values, derived for each species-sector combination, were allocated to each country based on the country’s share of the AOH for that species, calculated from the intersection of the species’ AOH map with each country’s borders^34^.

The nSTAR metric introduced here differs from the STAR metric from which it is adapted in that the normalisation step executed at this point in the STAR methodology is omitted. This ensures that the nSTAR metric is both additive and independent across all three dimensions of species, country, and economic sector, a necessary condition for use in input–output analysis. The STAR metric normalises the total value calculated in Eq. (2) to ensure that the total STAR value for any species is equal to *W*_*i*_ * 100, resulting in all species with the same extinction risk category being allocated the same STAR value regardless of the number of threats acting on them^26^. This normalisation facilitates the aggregation of the STAR metric by species taxonomy however it is problematic when aggregating the STAR metric by threat, since the STAR value attributed to each species-threat combination will be dependent not only on the characteristics of that threat, but also on the number and characteristics of other threats acting on the species. This dependence on more than one variable in the calculation of the STAR value for each species-threat combination means that it is not suitable for aggregation by threat and, by extension, economic sectors once the threat to sector allocation has been carried out.

In order to provide a metric which can be aggregated and disaggregated across species, sector, and country hierarchies the nSTAR methodology excludes this normalisation step. Consistent with the STAR methodology, the nSTAR metric is calculated using numeric values only and therefore has no unit of measure^26^.

### Input–output analysis

Once calculated, the nSTAR metric was partnered with the global supply-chain data available in the 2013 Eora MRIO, chosen for its extensive coverage of 190 regions (189 countries and one ‘rest of world’ region) and between 26 and 1022 economic sectors in each country, depending on the level of detail in each country’s publicly available National Accounts^12^.

A satellite block, or **Q** matrix, was created using the nSTAR values for 5295 species across 6357 economic sectors for 190 regions. This satellite block was then aggregated to match the sectoral structure of the Eora MRIO, a total of 14,839 country-sector combinations. A process flow diagram to illustrate the stages of data manipulation required to convert the IUCN Red List data to a satellite block ready for use with the Eora MRIO is included in Supplementary Fig. S5.

The Eora MRIO provided the intermediate transaction matrix **T,** the final demand matrix **Y**, and the value-added matrix **V**. Consumption based footprints were calculated by connecting the nSTAR value captured in the satellite block **Q** to the final demand matrix **Y** following Leontief’s methodology^9,10^. Central to this methodology is the Leontief Inverse **L**, a concise mathematical representation of the interdependencies across all economic sectors, which is expressed as:

3 $${\mathbf{L}} = \left( {{\mathbf{I}}{-}{\mathbf{A}}} \right)^{{ - {1}}}$$

where **I** is an identity matrix with dimensions equal to the those of the intermediate transaction matrix **T**, and **A** is the direct requirements matrix, derived from the **T** matrix in a number of stages. First the total output vector **x** is calculated, then diagonalised, and the inverse calculated to derive ${\widehat{\mathbf{X}}}^{-1}$, which returns the direct requirements matrix **A** when multiplied by **T.**

Next the satellite block was converted into an intensity matrix **q** by multiplying **Q** by ${\widehat{\mathbf{X}}}^{-1}$ to calculate the nSTAR value attributable to each dollar of total output produced by each sector. Once the **q**, **L** and **Y** matrices are available, the consumption extinction-risk footprint for a sector *k* (**f**_*k*_) can be calculated using Eq. (4):

4 $${\mathbf{f}}_{k} = {\mathbf{q}}*{\mathbf{L}}*{\mathbf{Y}}_{k}$$

where **Y**_*k*_ represents the final demand for that sector. Consumption extinction-risk footprint values were generated for each species-sector-country combination, a total of more than 78 million datapoints.

Further matrix manipulation was used to calculate the country-level imported, exported, and domestic extinction-risk footprints. For each country the final demand matrix, **Y**, was separated into two matrices, **Y**_*dom*_, representing demand from that country for the economic sectors in that country, and **Y**_*oth*_, representing demand from all other countries for the economic sectors in that country. Next, the intensity matrix, **q**, was separated into two matrices, **q**_*dom*_, representing the nSTAR intensity for each of the species within that country’s borders, and **q**_*oth*_, representing the nSTAR intensity for all remaining species. The three sub-footprints for each country were calculated using Eqs. (5), (6) & (7). A simplified illustration of this methodology is included in Supplementary Fig. S3.

5 $${\mathbf{f}}_{dom} = {\mathbf{q}}_{dom} *{\mathbf{L}}*{\mathbf{Y}}_{dom}$$

6 $${\mathbf{f}}_{exp} = {\mathbf{q}}_{dom} *{\mathbf{L}}*{\mathbf{Y}}_{oth}$$

7 $${\mathbf{f}}_{imp} = {\mathbf{q}}_{oth} *{\mathbf{L}}*{\mathbf{Y}}_{dom}$$

Imported, exported, and domestic extinction-risk footprints were calculated for 188 countries.

### Limitations

While very powerful in unravelling the intricacies of the global economy, there are limitations to the effectiveness of input–output analysis. Since it relies on National Accounts data, only activity which can be directly connected into reported economic activity is captured. This means that any activities that are not transacted within the boundaries of the formal economy, such as subsistence hunting and illegal logging, will be excluded unless they have been incorporated into the relevant country’s National Accounts data. The exclusion of threats due to their timing or non-economic classification (such as geological events, disease, and invasive species) resulted in a zero nSTAR value for 519 species, leaving 4776 species with a material nSTAR value. In addition, any limitations in the sector categorisations, their spatial and technological homogeneity, or assumptions included in the allocation of economic activity to sectors within the National Accounts data in each country will be propagated through to the footprint calculations. These limitations are common to consumption-based analyses^5–7,17,25^ and we do not further address them here.

Further limitations exist with the use of the scope and severity data for each threat captured in the IUCN Red List, since this does not take into account interaction between threats, or between the severity and scope of an individual threat^36^. As a result, the impact from a single threat acting on a species may be overstated, and higher nSTAR values attributed to that species than would otherwise be warranted. In addition, any variations in the location, scope, or severity of threats acting across a species’ distribution range are not captured and thus the impact of different economic sectors may be over or under-represented^26^.

There is a temporal displacement between the economic activity and the extinction risk used in this analysis. The extinction risk category assigned to each species is due to the cumulative sum of current and historical impacts acting on it, while the value of economic interactions used to trace this extinction risk through the global economy is based on one year of activity. This is typical of related approaches^1,6^, and may not introduce much uncertainty given that current economic activity is higher than at any time in history^37^. Nevertheless, there is no doubt that some current extinction risk is due to past economic activity and development of methods to incorporate this temporal dimension would be a valuable research avenue.

## Supplementary Information

Supplementary Information.

## Data Availability

Datasets generated in this study have been deposited to the following repositories: Dataset 1: https://doi.org/10.25910/smtv-e036. Dataset 2: https://doi.org/10.25910/e60m-2r62. Dataset 3: https://doi.org/10.25910/n1sd-rw69. Eora 2013, including details of the sector categorisation used in each country, is available at www.worldmrio.com. IUCN Red List of Threatened species is available at: https://apiv3.iucnredlist.org/. Sector to threat concordance is available at: https://hdl.handle.net/2123/24233**.**
