# Supplementary material for: Quantifying and categorising national extinction-risk footprints
Source: Sci Rep. 2022 Apr 7;12:5861. doi: 10.1038/s41598-022-09827-0 (PMC8991243; doi:10.1038/s41598-022-09827-0)
Supplement: Supplementary file 1 — Supplementary Information. [file 41598_2022_9827_MOESM1_ESM.pdf]

Supplementary Information

Quantifying and categorising national extinction-risk footprints

Amanda Irwin*, Arne Geschke, Thomas M. Brooks, Juha Siikamaki, Louise Mair, Bernardo B. N. Strassburg

Supplementary Information

This Supplementary Information includes:

S1	Illustration of flow of direct impact by sector to indirect impact by sector	Page 2
S2	Further information on characteristics of each country's extinction-risk footprint	Page 3
S3	Country share of extinction-risk footprint for selected species	Page 5
S4	Extinction-risk footprint by sector and taxonomic group	Page 6
S5	Supplemental Methods	Page 7
	Connection of economic sectors to IUCN Threats Classification Scheme<br>Illustration of data manipulation steps to create an Eora-compatible satellite block	
Supplementary References		Page 9

S1 - Illustration of flow of direct impact by sector to indirect impact by sector

Input-output analyses consider the flow of money transacted through complex supply chains which connect raw materials to the final point of consumption, via a multitude of intermediate transactions. Any environmental impact associated with the activity at each node of these supply chains can also be analysed and attributed to the same final consumption. In this analysis we connect the direct extinction risk impact from each source sector to the indirect extinction risk impact, or consumption extinction-risk footprint, generated by consumption at the point of final demand.

A simplistic illustration of the transformation from direct impact to indirect impact through this analysis is found in Figure S1. The sectors along the left-hand side have directly contributed to the threats acting on each species, with the impact quantified as outlined in Methods. This impact then ‘flows’ to the consumption sectors on the right-hand side, through the trade of products and services and proportionate to the final expenditure in each of these consumption sectors, generating the consumption extinction-risk footprint of each sector.

Take, for example, the impact created by the dark green coded agriculture sector, contributing 54% of the direct, production-based, extinction risk but only 19% of the indirect, consumption-based, extinction risk. This direct impact ‘flows’ to other sectors as money is spent to consume the products and services provided by the agriculture sector, with 37% ‘flowing’ to food & beverage (light blue), 6% to construction (purple) and 34% to final demand for agriculture itself. For simplicity, the multitude of intermediate interactions which take place between the direct activity and final consumption have not been included in this diagram.

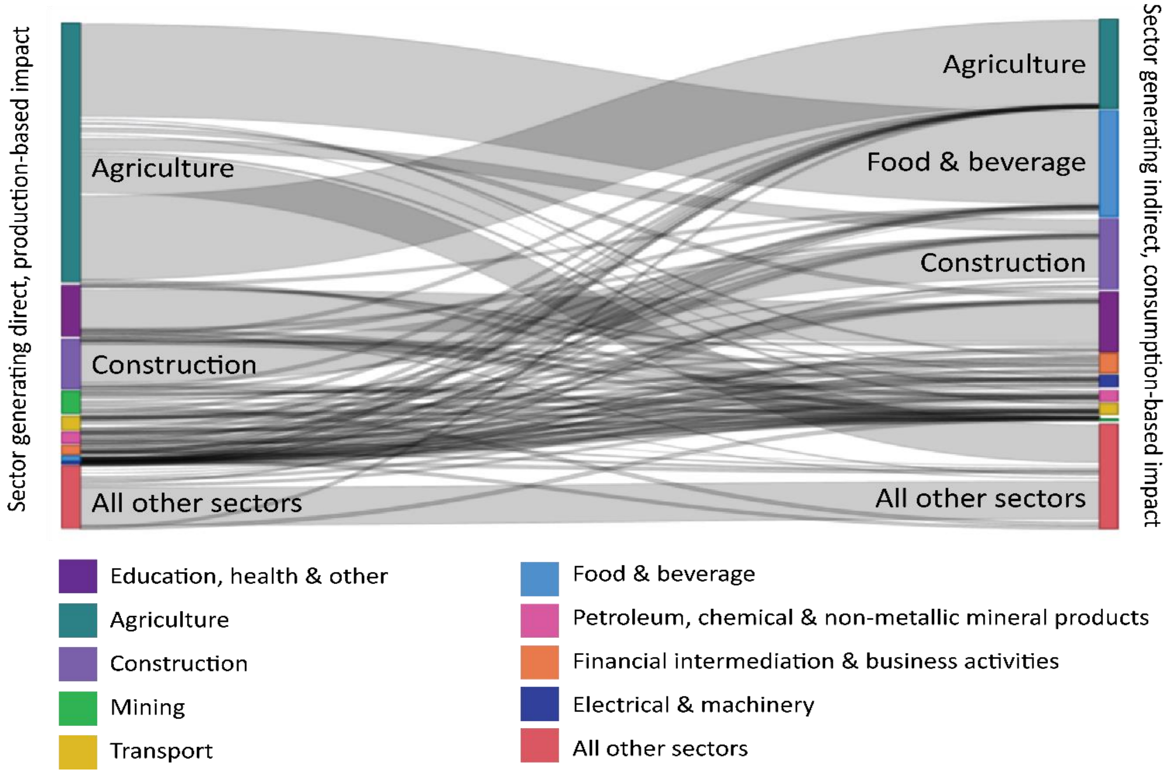

Figure S1. Extinction risk flow from source of activity to source of consumption. The sector responsible for direct activity is on the left, the consumption sector is on the right. Intermediate interactions have been excluded for simplicity (Generated using Microsoft PowerBI https://powerbi.microsoft.com/en-us/power-bi-pro/)

S2 - Further information on characteristics of each country's extinction-risk footprint

A country's total consumption extinction-risk footprint represents the impact of that country's consumption on species located in all countries, while its total territorial extinction-risk footprint represents the impact of global consumption on the species found within that country's borders. We further refine these footprints by considering different combinations of the location of both consumption and extinction risk to define a domestic, imported, and exported footprint (see Methods). Figure S2 provides a visual representation of the breakdown of the territorial and consumption extinction-risk footprints for Australia, as an example.

A country's domestic extinction-risk footprint occurs when consumption within the country drives extinction risk within the country - in the case of Australia this represents 78% of the consumption footprint. The imported extinction-risk footprint is a result of consumption within the country driving extinction risk in other countries (22% of Australia's consumption footprint), and its exported extinction-risk footprint is a result of consumption outside of the country driving the extinction risk within the country (38% of Australia's territorial footprint). The consumption footprint for each country can be calculated by adding the domestic and imported footprints, and the territorial footprint can be calculated by adding the domestic and exported footprints. At a global level, and at a total species level, the total consumption extinction-risk footprint will equal the total territorial extinction-risk footprint.

Input-output analysis is used to calculate these specific footprints by adjusting the satellite matrix **(Q)** and final demand matrix **(Y)** so that only values for the country or countries in question are included (see Methods). A visual representation of these calculations is found in Figure S3, which illustrates a simple 5 country economy with a 5x5 Leontief Inverse **(L)** matrix, a 5x5 final demand **(Y)** matrix, and a 1x5 satellite **(Q)** matrix.

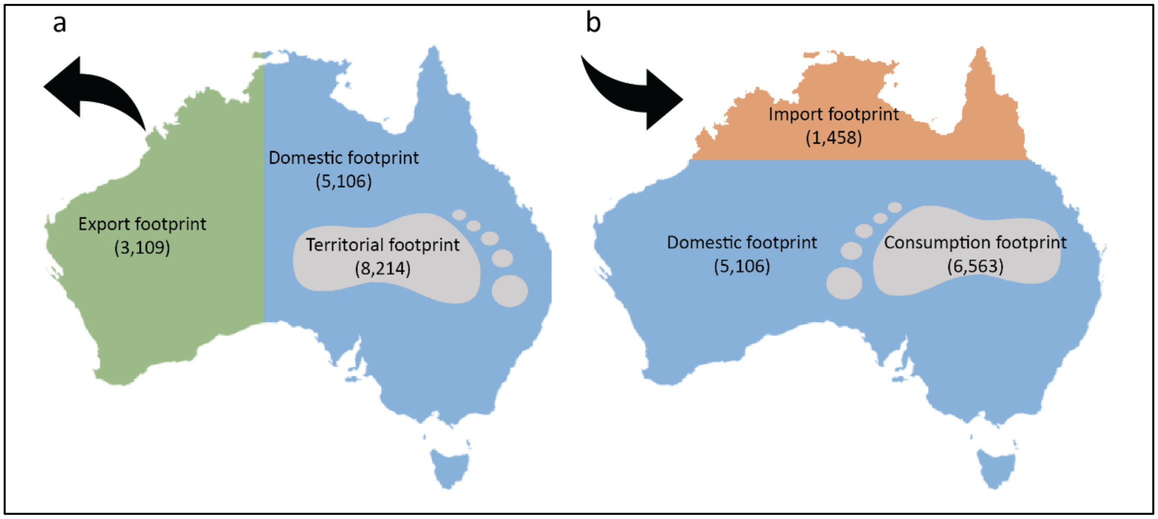

Figure S2. Australia's extinction-risk footprints. A visual representation of the different footprint categories for Australia: (**a**) Australia's territorial footprint is made up of its domestic and exported footprints; (**b**) Austraila's consumption footprint is made up of its domestic and imported footprints. (Generated using Microsoft PowerPoint https://www.microsoft.com/en-us/microsoft-365/powerpoint)

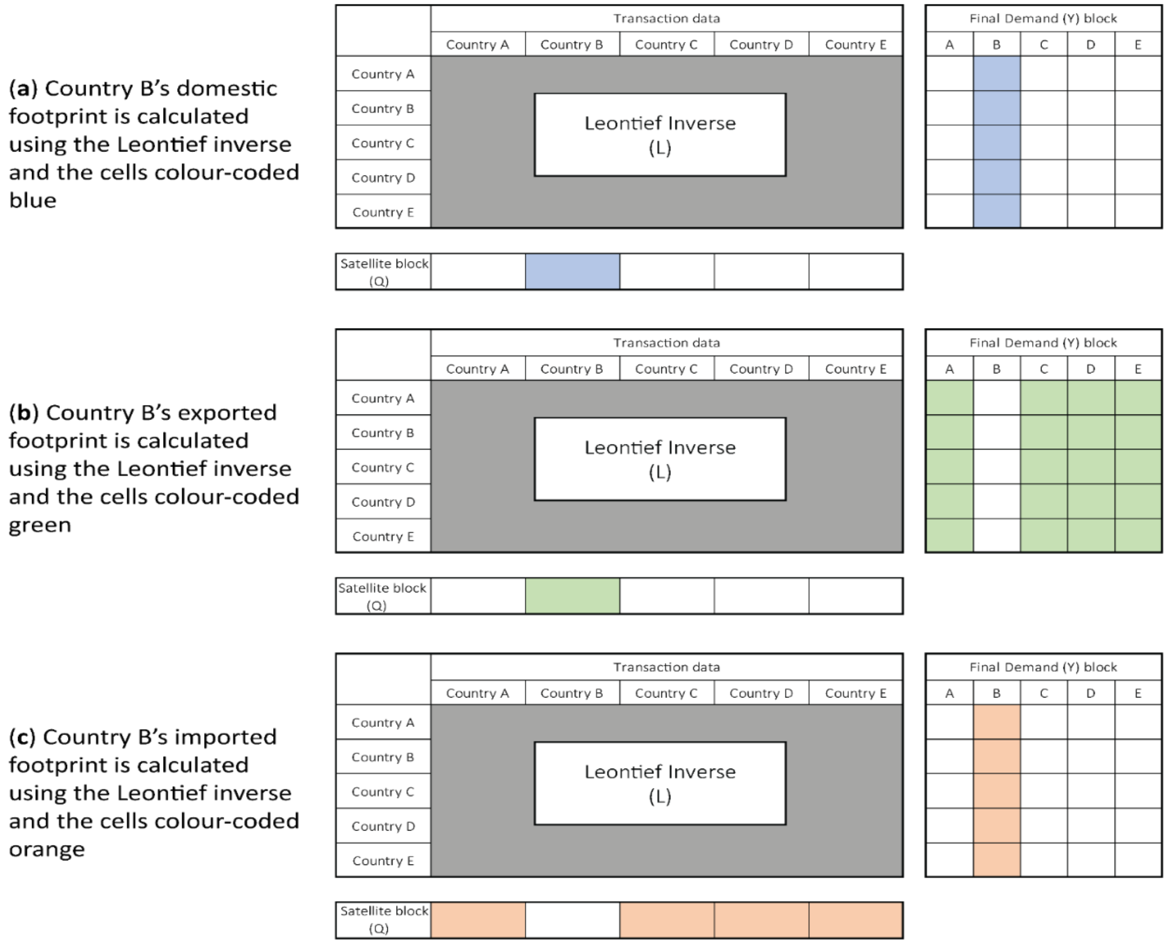

Figure S3. A simplified illlustration of the use of input-output analysis to calculate three footprints for each country. The Leontief Inverse (**L**) matrix contains information on the economic interdependencies in this simple 5 country economy, which along with a 5x5 final demand (**Y**) matrix, and a 1x5 satellite (**Q**) matrix, can be used to calculate 3 different footprints for each country: (**a**) the domestic footprint; (**b**) the exported footprint and; (**c**) the imported footprint.

S3 - Country share of extinction-risk footprint for selected species

The contribution that consumption based in each country makes to the extinction-risk footprint of three selected species is illustrated in Figure S4. Domestic consumption is based in the country/countries where the species is present. Only countries which contribute more than 1% of the species' extinction-risk footprint have been highlighted.

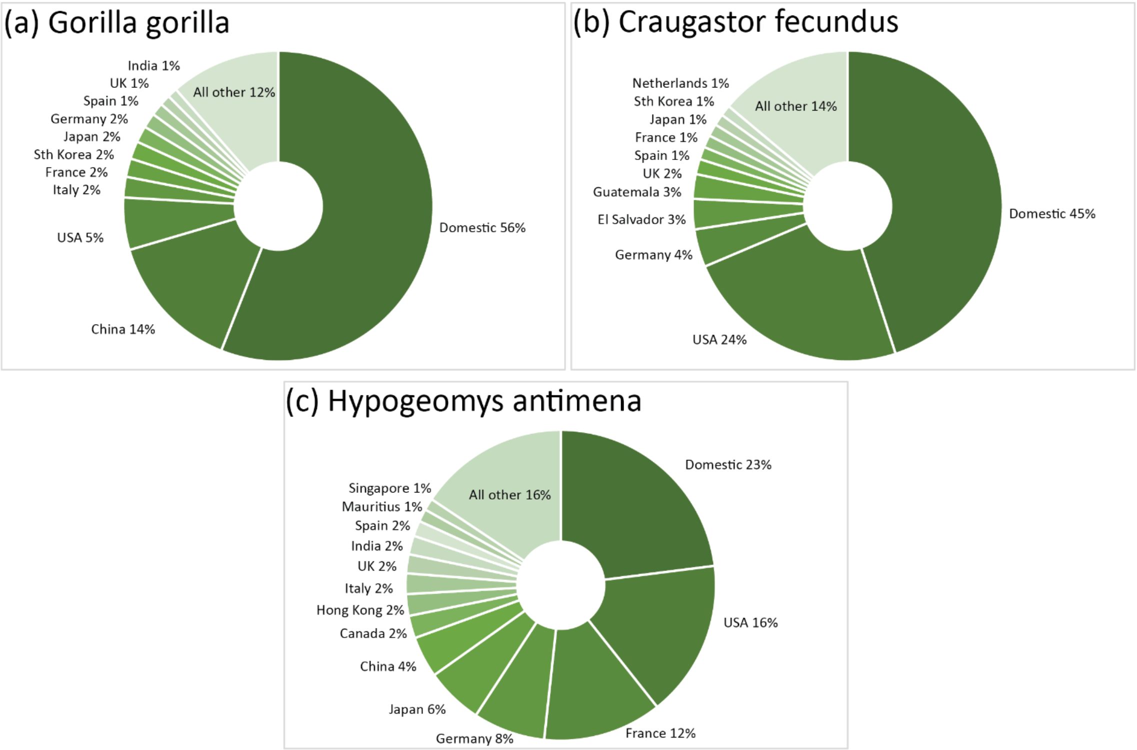

Figure S4. Country share of extinction-risk footprint for selected species: (**a**) *Gorilla gorilla*, found in western Africa; (**b**) *Craugastor fecundus*, found in Honduras; (**c**) *Hypogeomys antimena*, found in Madagascar. Only countries which contribute more than 1% of the species' extinction-risk footprint have been highlighted. (Generated using Microsoft Excel https://www.microsoft.com/en-au/microsoft-365/excel)

S4 - Extinction-risk footprint by sector and taxonomic group

Each aggregated economic sector's contribution to the global extinction-risk footprint is included in Table S1, for all species in scope, and for each Class.

Table S1: Share of extinction-risk footprint by sector at Class level

Sector	All in-scope species	Amphibians	Mammals	Birds
Food & beverage	20.2%	20.8%	17.8%	21.9%
Agriculture	19.3%	19.0%	19.1%	20.0%
Construction	15.9%	17.9%	14.9%	14.3%
Financial intermediation & business activities	8.6%	9.5%	8.6%	7.2%
Education, health & other services	6.2%	7.0%	6.3%	5.2%
Electrical & machinery	4.0%	3.2%	4.5%	4.8%
Transport equipment	3.2%	2.1%	4.6%	3.3%
Petroleum, chemical & non-metallic mineral products	2.7%	2.6%	2.6%	2.9%
Hotels & restaurants	2.3%	2.1%	2.4%	2.4%
Retail trade	2.1%	1.9%	2.3%	2.1%
Transport	2.1%	2.0%	2.3%	1.9%
Wholesale trade	2.0%	1.8%	2.1%	2.1%
Public administration	1.9%	1.6%	2.3%	1.8%
Textiles & wearing apparel	1.9%	1.9%	1.7%	1.9%
Electricity, gas & water	1.2%	1.2%	1.2%	1.2%
Others	1.2%	1.1%	1.3%	1.2%
Other manufacturing	1.1%	0.7%	1.4%	1.4%
Post & telecommunications	1.0%	0.9%	1.2%	1.0%
Mining	1.0%	0.9%	0.9%	1.1%
Metal products	0.6%	0.6%	0.7%	0.7%
Wood & paper	0.6%	0.6%	0.7%	0.6%
Recycling	0.4%	0.2%	0.6%	0.4%
Fishing	0.3%	0.2%	0.3%	0.5%
Re-export & re-import	0.1%	0.1%	0.1%	0.1%
Maintenance & repair	0.1%	0.1%	0.1%	0.1%
Private households	< 0.1%	< 0.1%	< 0.1%	< 0.1%
TOTAL	100%	100%	100%	100%

S5 - Supplementary Methods

Connection of economic sectors to IUCN Threats Classification Scheme

The allocation of each economic sector within the UN Statistics Division Central Product Classification Standard[1] to relevant IUCN Threat classifications[2] created a 6357 x 118 concordance matrix, which was then weighted based on the size of each of the economic sectors. The weighting for each sector-threat combination was used to allocate the nSTAR value for each species-threat combination to the sectors which are likely to generate activity directly related to that threat.

Consider for example the two sectors related to cows:

010210 – Bovine, live pure-bred breeding

010290 – Bovine, live except pure-bred breeding

Both of these sectors can be connected into Threats 2.3.2, 2.3.3 and 2.3.4 which cover grazing, ranching, or farming on different scales, however pure-bred breeding is unlikely to be connected to Threat 2.3.1 – Nomadic grazing. The sector to threat concordance connects sector 010290 into Threats 2.3.1, 2.3.2, 2.3.3, and 2.3.4 but only connects sector 010210 into Threats 2.3.2, 2.3.3 and 2.3.4.

The full sector to threat concordance matrix is available at: https://hdl.handle.net/2123/24233

Illustration of data manipulation steps to create an Eora-compatible satellite block

Figure S5 provides a process flow diagram to illustrate the stages of data manipulation required to convert the IUCN Red List data to a satellite block ready for use with the Eora MRIO.

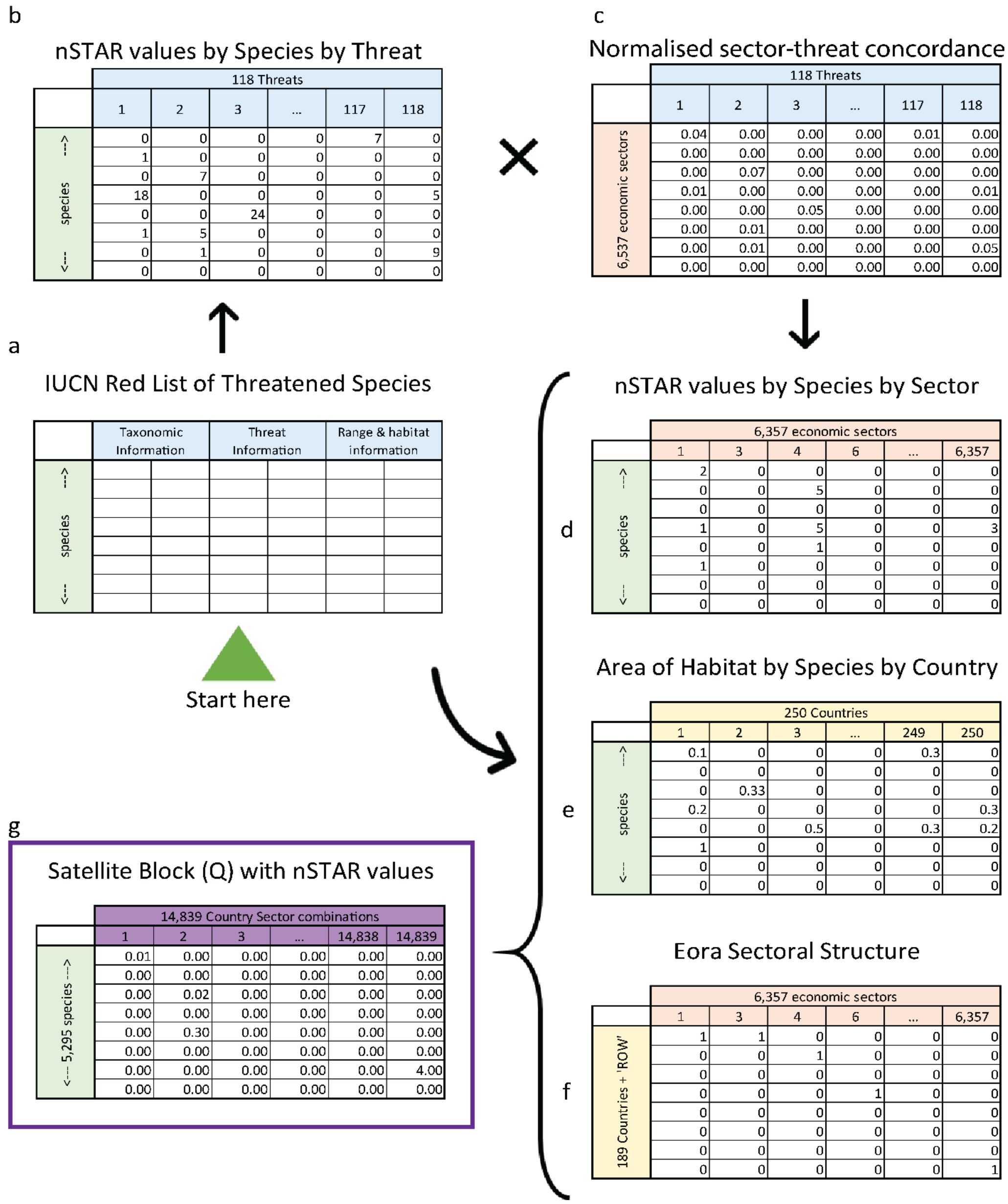

Figure S5. Flow of data manipulation to convert IUCN Red List data to an Eora-compatible satellite block: (**a**) Information is retrieved from the IUCN Red List of Threatened Species for all species in scope[3]; (**b**) Information related to the threats acting on each species is used to generate nSTAR values for that species as per Methods; (**c**) A concordance which connects each economic sector to the 118 threats in the IUCN Threats Classification Scheme[2] is weighted by the size of each economic sector to generate a sector to threat matrix; (**d**) This matrix is multiplied by the nSTAR values for each species-threat combination in (b) to calculate the nSTAR value for each species-sector combination; (**e**) An area of habitat map is generated for each species[4]; (**f**) The sectoral structure of the Eora MRIO[5] is obtained; (**g**) The outputs of (d), (e), and (f) are used to generate the final satellite block.

Supplementary References

1 United Nations Statistics Division. *Central Product Classification (CPC) Version 2.1*, <https://unstats.un.org/unsd/classifications/unsdclassifications/cpcv21.pdf> (2015).

2 IUCN. *Threats Classification Scheme (Version 3.2)*, <https://www.iucnredlist.org/resources/threat-classification-scheme> (2020).

3 IUCN. *The IUCN Red List of Threatened Species. Version 2020-2*, <https://www.iucnredlist.org/> (2020).

4 Strassburg, B. B. N. *et al.* Global priority areas for ecosystem restoration. *Nature* **586**, 724-729, doi:10.1038/s41586-020-2784-9 (2020).

5 Lenzen, M., Moran, D., Kanemoto, K. & Geschke, A. Building Eora: A global multi-region input-output database at high country and sector resolution. *Economic Systems Research: Global Multiregional Input-Output Frameworks* **25**, 20-49, doi:10.1080/09535314.2013.769938 (2013).
